# Characterization of Conjunctival Sac Microbiome from Patients with Allergic Conjunctivitis

**DOI:** 10.3390/jcm11041130

**Published:** 2022-02-21

**Authors:** Hang Song, Kang Xiao, Hanyi Min, Zhengyu Chen, Qin Long

**Affiliations:** 1 Department of Ophthalmology, Peking Union Medical College Hospital, Beijing 100730, China; songhang@pumch.cn (H.S.); pumc_xk@student.pumc.edu.cn (K.X.); minhy@pumch.cn (H.M.); pumc_chenzhengyu@student.pumc.edu.cn (Z.C.); 2 Key Laboratory of Ocular Fundus Diseases, Chinese Academy of Medical Sciences & Peking Union Medical College, Beijing 100730, China

**Keywords:** conjunctival sac, microbial diversity, microbial composition, allergic conjunctivitis

## Abstract

Conjunctival sac microbiome alterations have been reported to be closely associated with many ocular diseases. However, the characteristic of conjunctival sac microbiome in allergic conjunctivitis (AC) was scarcely described. In this study, we aimed to identify the differences of the conjunctival sac microbiome composition in AC patients compared with normal controls (NCs) using high-throughput 16S rDNA sequencing metagenomic analysis. The conjunctival sac microbiome samples from 28 AC patients and 39 NC patients were collected. The V3-V4 region of 16S rRNA gene high-throughput sequencing was performed on the illumina MiSeq platform. Alpha diversity, beta diversity and the relative abundance at the phylum and genus levels were analyzed using QIIME. Alpha diversity demonstrated by Chao1, Observed_species and PD_whole_tree indexes did not show significant difference between the AC and NC groups, while the Shannon index was higher in the AC group. Beta diversity showed divergent microbiome composition in different groups (*p* < 0.005). The top five abundant phyla were Firmicutes, Proteobacteria, Actinobacteriota, Bacteroidota and Cyanobacteria in both groups. The top five abundant genera were *Bacillus, Staphylococcus*, *Corynebacterium, Acinetobacter* and *Ralstonia* in the AC group and *Acinetobacter*, *Staphylococcus*, *Bacillus*, *Clostridium_sensu_stricto_1*, *Corynebacterium* and *Geobacillus* in the NC group. The Firmicutes/Bacteroidetes (F/B) ratio at the phylum level was similar between groups (*p* = 0.144). The *Bacillus/Acinetobacter* (*B/A*) ratio at the genus level was higher in the AC group (*p* = 0.021). The dysbiosis detected in this study might provide further evidence to investigate the mechanism and treatment methods for allergic conjunctivitis.

## 1. Introduction

Allergic conjunctivitis (AC) is a common and potentially debilitating ocular surface disease characterized by antigen-specific immunoglobulin E (IgE) and T helper type 2 (Th2) lymphocyte–mediated type I hypersensitivity [1,2]. The sensitization process begins when antigen-presenting cells in the conjunctiva present antigens to naive T cells. The naive T cells mature into Th1 and Th2 lymphocytes, which secrete cytokines that promote B-cell differentiation and IgE production [3]. When next time an allergen is detected, IgE activates mast cells, resulting in the release of preformed mediators promoting vasodilation, vascular permeability, smooth muscle contraction and inflammatory cell recruitment. Patients with AC usually present with itching, redness and swelling of the conjunctiva [4].

There have been studies showing that antibiotic use in early life is positively associated with the development of various allergic diseases; a possible hypothesis is that dysbiosis during immune system maturation might contribute to the development of allergic disease [5,6,7]. There have been extensive studies showing that gut microbiota alterations are associated with various diseases, not only in the gut, such as inflammatory bowel disease [8], irritable bowel syndrome [9] and celiac disease [10], but also systemically such as rheumatoid arthritis [11,12], systemic lupus erythematosus [13,14] spondylarthritis [15,16], primary Sjögren’s syndrome [17], multiple sclerosis [18] and Behcet’s disease [19]. The dysbiosis of gut microbiota could also regulate the immune system and activate certain immune cells that cause diseases in certain organs [20]. Specifically for the gut–eye axis, gut microbiota alterations have been shown in patients with Behcet’s uveitis [19], Vogt–Koyanagi–Harada disease [21] and keratitis [22].

While gut microbiota regulate the immune response in remote organs, previous studies have also shown that dysbiosis of the microbiota in other areas such as the skin and the airways might as well play an important role in allergic diseases locally like atopic dermatitis and asthma via influencing immune responses [23,24,25]. For ocular surface, conjunctival sac microbiome alterations were reported to be closely associated with several diseases, such as blepharitis, dry eye, Stevens–Johnson syndrome, and keratitis [26,27,28,29,30,31,32,33]. Hence, we hypothesized that the dysbiosis of conjunctival microbiome is associated with AC.

In this study, we aimed to identify the differences of the conjunctival sac microbiome between AC patients and healthy subjects using high-throughput 16S rDNA sequencing metagenomic analysis, which could identify a much more diverse microbiota that might not be recovered by conventional culture methods [34]. To date, this is the first study to focus on the association between conjunctival sac microbiota and AC using the 16S rDNA method. 

## 2. Materials and Methods

### 2.1. Sample Collection

Patients were recruited from the outpatient service, Department of Ophthalmology, Peking Union Medical College Hospital between 1 September and 30 October 2021. Patients with allergic conjunctivitis who were treatment naïve were included in this study. Allergic conjunctivitis was diagnosed based on symptoms (itching, foreign body sensation and increased ocular discharge in the conjunctival sac), clinical signs (conjunctival hyperemia, conjunctival papilla and specific corneal lesions changes) and identification of specific allergens. Patients with healthy conjunctival sac conditions were included as the NC group after ruling out other ocular surface illness on slit lamp examination. For both groups, patients with systemic diseases, history of any kind of oral drugs and eye drop usage within the past 2 months were excluded from this study. A sterile cotton swab was used to collect specimens by rubbing the swab from the medial to the lateral side of inferior fornix of the conjunctival sac of each right eye without anesthesia. The swabs were then placed in sterile tubes and stored in a refrigerator (at −20 °C) before further experiments. The study adhered to the tenets of the Declaration of Helsinki and was approved by the Institutional Review Board of Peking Union Medical College Hospital (ZS-3092). Informed consent forms were obtained from all patients.

### 2.2. DNA Extraction, PCR Amplification, and 16S rRNA Gene Amplicon Sequencing

DNA was extracted using the MicroElute Genomic DNA Kit (D3096, Omega, MA, USA) according to the manufacturer’s instructions. The concentration of DNA was measured using a NanoDrop 2000 ultramicro-spectrophotometer (Thermo Scientific, Waltham, MA, USA). The V3–V4 region of the 16S rRNA gene was amplified from extracted genomic DNA samples and the primers (319 F: 5′-ACTCCTACGGGAGGCAGCAG-3′ and 806 R: 5′-GGACTACHVGGGTWTCTAAT-3′). PCR was carried out on a Mastercycler Gradient (Eppendorf, Germany) using 25 μL reaction volumes, containing 12.5 μL 2× Taq PCR MasterMixⅡ (Vazyme Biotech Co., Ltd., Nanjing, China), 3 μL BSA(2 ng/μL), 1 μL Forward Primer (5 μM),1 μL Reverse Primer (5 μM), 2 μL template DNA and 5.5 μL ddH_2_O. The PCR amplification products were purified with Agencourt AMPure XP magnetic beads (Fisher Scientific, Hampton, NH, USA), dissolved in elution buffer and then labeled. The fragment range and the concentration of the library were detected using the Agilent 2100 Bioanalyzer (Agilent, Santa Clara, CA, USA). Qualified libraries were selected for sequencing on the MiSeq PE300 platform based on the size of the inserted fragments.

### 2.3. Bioinformatics Analysis

Samples were sequenced on an illumina MiSeq platform (LC-Bio) according to the manufacturer’s instructions. Qualified paired-end reads were separated using the sample-specific barcode sequences and trimmed with Illumina Analysis Pipeline Version 2.6. Paired-end Reads were screened by Pear (v0.9.6) software and removed from consideration if they were shorter than 120 bp, had a low-quality score (≤20) and contained ambiguous bases. The qualified sequences were clustered into operational taxonomic units (OTUs) at a similarity ≥ 97% use Uparse algorithm of Vsearch (v2.7.1) software, to generate rarefaction curves and to calculate the richness and diversity indices. The Ribosomal Database Project (RDP) Classifier tool was used to classify all sequences into different taxonomic groups against Silva138 database.

Alpha diversity was employed to analyze complexity of species diversity for each sample through Chao1, Observed_species, PD_whole-tree and Shannon indexes generated by QIIME (Version 1.8.0; Boulder, CO, USA). Beta diversity was demonstrated by the principal co-ordinates analysis (PCoA) and Partial Least Squares Discrimination Analysis (PLS-DA) to evaluate microbiome complexity between samples. The taxonomy and relative abundance were bioinformatically analyzed at phylum, genus and species levels.

### 2.4. Statistical Analysis

The R software (Version 3.2.5; Auckland, New Zealand) and GraphPad prism 5.0 (GraphPad Software, San Diego, CA, USA) were used for statistical analyses. Tukey’s test was used to identify significant between-group differences for alpha-diversity. The divergence between two groups was compared by ANOSIM analysis. The relative abundance of bacteria was compared by the one-way analysis of variance. The age comparison was presented as mean ± standard deviations (SD) and examined by Student’s *t* test. Sex distribution was presented as proportions and examined by Chi-square test. *p-*value less than 0.05 was considered statistically significant.

## 3. Results

### 3.1. Demographic Characteristics of Patients

Conjunctival sac microbiome samples were collected from 28 eyes of patients diagnosed with AC and 39 eyes of patients with healthy ocular surface conditions. The 39 normal control patients included 33 patients diagnosed with refractive error, two patients with cataract and four patients without ocular diseases. The overall subjects included 20 (29.85%) males and 47 (70.15%) females with age ranging from 13 to 62 years old (33.33 ± 11.53). The mean age of patients in the allergic conjunctivitis group and control group was 31.69 and 35.61, respectively (*p* = 0.847) (Table 1). 

### 3.2. NGS Data

A total of 3,641,254 high-quality sequences were generated from 67 samples with an average of 54,347 sequences per sample. High-quality sequences were clustered into 3428 Operational Taxonomic Units (OTUs) at 97% sequence identity. A subsampled OTU table was obtained consisting of 3383 OTUs (ranging from 31 to 609 per sample), corresponding to 867 genera, 441 families, 264 orders, 117 classes and 42 phyla. A total of 1676 common OTUs were identified, representing 57.34% (961/1676) and 44.51% (746/1676) of the total OTUs in the AC and NC group, respectively.

### 3.3. Alpha Diversity

The rarefaction curve flattened as the number of reads increased (Figure S1), with Goods coverage values all close to 1 (≥0.99, all samples), indicating that all the samples were sufficiently sequenced to represent its identity. The species accumulation curve flattened with the number of samples sequenced (Figure S2), demonstrating that the sample size is adequate to represent the overall bacterial diversity in the targeted population.

**Figure 1:**
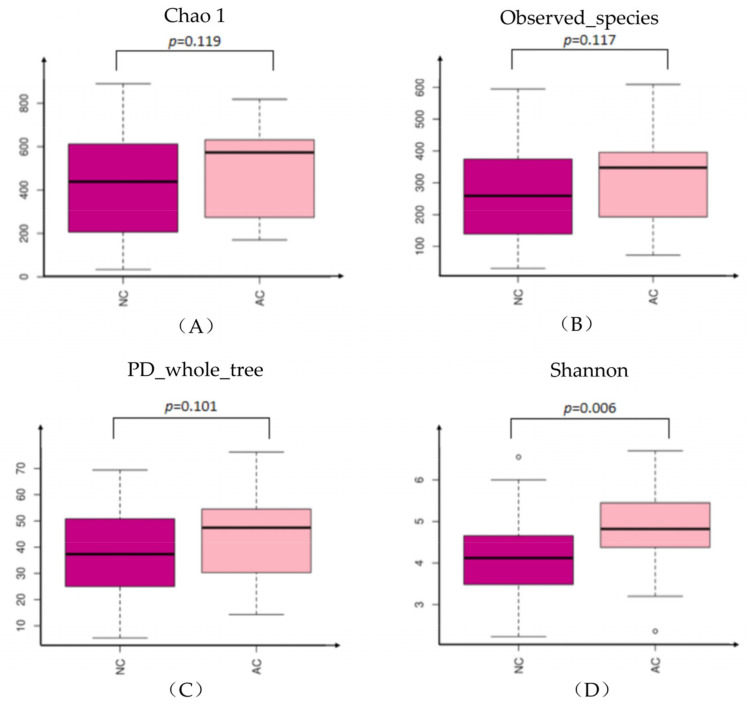
The alpha diversity represented by Chao1 (**A**), Observed_species (**B**), PD_whole-tree (**C**) and Shannon (**D**) indexes. NC: normal control, AC: allergic conjunctivitis, PD: phylogenetic diversity.

The alpha diversity was represented by Chao1, Observed_species, PD_whole-tree and Shannon indexes. Chao1, Observed_species, PD_whole-tree indexes did not show significant difference between the two groups, while the Shannon index was statistically significant higher in the AC group (Figure 1). Sex and age were further analyzed in each subgroup, but revealed no statistically significant difference (data not shown). 

### 3.4. Beta Diversity

**Figure 2:**
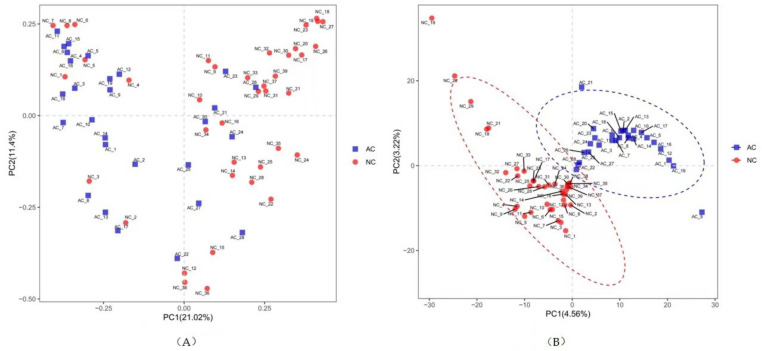
The beta diversity represented by weighted PCoA (**A**) and PLS-DA (**B**). NC: normal control, AC: allergic conjunctivitis, PC: principal component, PCoA: principal co-ordinates analysis), PLS-DA: Partial Least Squares Discrimination Analysis.

Phylogenetic variation of the microbial communities was analyzed with beta diversity indices as demonstrated by the weighted PCoA and PLS-DA. Samples in the AC group were more centralized and resembled each other in bacterial composition, while samples in the NC group were more acentric and disperse (Figure 2). There was significant divergence between the AC and NC groups by ANOSIM analysis (R = 0.199, *p* = 0.0001). 

### 3.5. Bacteria Predominance

**Figure 3:**
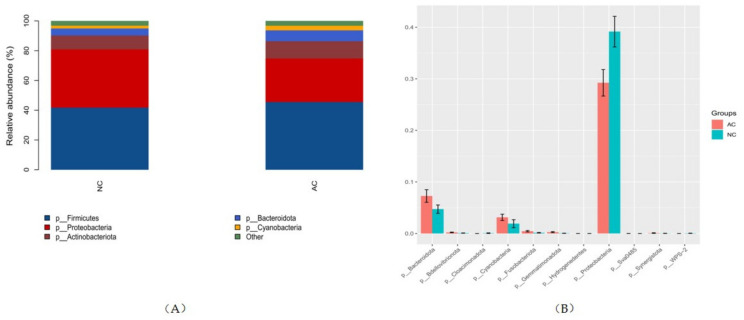
The top five most abundant phyla in each group (**A**) and phyla that were statistically different in abundance (**B**). NC: normal control, AC: allergic conjunctivitis, p: phylum.

We summarized the relative abundance of the dominant bacterial community in each group of patients. At the phylum level, 39 phyla were detected from the AC group and 41 phyla were detected from the NC group. The top five most abundant phyla were Firmicutes, Proteobacteria, Actinobacteriota, Bacteroidota and Cyanobacteria in both groups (Figure 3). However, the relative abundances of the top five phyla were relatively different (Table 2). There were 11 statistically different abundant phyla (*p* ≤ 0.05) in total (Figure 3). The Firmicutes/Bacteroidetes (F/B) ratio, which was correlated with inflammation in gut microbiome studies, was also calculated. The F/B ratio was 37.67 ± 12.85 and 15.16 ± 4.12 in the AC and NC group, respectively (*p* = 0.144). 

**Figure 4:**
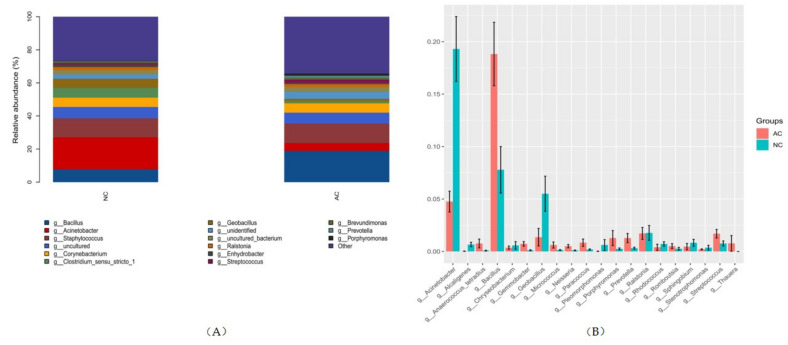
The top five most abundant genera in each group (**A**) and phyla that were statistically different in abundance (**B**). NC: normal control, AC: allergic conjunctivitis, g: genus.

At the genus level, there were 743 genera detected in the AC group with the top five abundance order of *Bacillus, Staphylococcus*, *Corynebacterium, Acinetobacter* and *Ralstonia*. There were 718 genera detected in the NC group with the top five abundance order of *Acinetobacter*, *Staphylococcus*, *Bacillus*, *Clostridium_sensu_stricto_1*, *Corynebacterium* and *Geobacillus* (Figure 4). There were 249 genera that were statistically different in their relative abundance in these two groups (*p* ≤ 0.05). Of these, the top 20 remarkable and abundant genera are shown in Figure 4, with *Acinetobacter* and *Bacillus* contributing the most significant difference. The Bacillus/Acinetobacter (B/A) ratio was 16.98 ± 5.36 and 4.70 ± 2.11 in the AC and NC group, respectively (*p* = 0.021).

At the species level, the most predominant three bacteria are *Bacillus_sp, Staphylococcus_epidermidis* and *Acinetobacter_guillouiae*; the relative abundances were 18.28%, 11.86% and 2.75% in the AC group and 7.50%, 11.51% and 27.95% in the NC group, respectively.

## 4. Discussion

In recent years, evidence has been accumulating that the microbiota in microenvironments is important in the defense against pathogens and the maintenance of homeostasis [35]. Intensive attention has been focused on the gut microbiome system, which has been shown to be correlated with many systemic diseases and even some ocular diseases such as dry eye [31,36,37,38], uveitis [19,21] and keratitis [22]. While the ocular surface is considered paucibacterial [39], there have been studies demonstrating that microbiota-dependent ocular defense systems could defend against pathogens by differentiating pathologic and commensal organisms. For example, Cornebacterium mastitidis was found to induce IL17 production in the ocular mucosa and confer protection Candida albicans or Pseudomonas aeruginosa infection [40] on the conjunctival sac in mice studies.

Alpha diversity is the average species diversity in a particular sample and is also termed as local diversity. In this study, alpha diversity represented by Chao1, Observed_species and PD_whole_tree indexes did not show significant difference between the AC and NC groups. However, the Shannon index, which provides information about both richness and evenness, was higher in the AC group. Previously, a study regarding ocular microbiome studies in allergic rhinoconjunctivitis also showed that the alpha diversity was not significantly changed in allergic rhinoconjunctivitis. However, in that study, the Shannon index of ocular samples decreased with disease clinical severity scores [41]. This minor discrepancy in the Shannon index could not yield potent conclusions whether the Shannon index was changed in patients with AC. Combined with other parameters, the alpha diversity was still preferred to be considered stable and unchanged in patients with AC.

Beta diversity is the measure of dissimilarity between two groups. In this study, we revealed different microbiome composition by weighted PCoA, PLS-DA analysis. Samples in the AC group were more centralized and resembled each other in bacterial composition, which might indicate that microbiota in the AC group has a tendency to share similar characteristics. This differentiation was further confirmed by ANOSIM analysis, which showed the difference between the groups was greater than the difference within the group.

There have been studies showing that the most predominant phyla were Actinobacteria in the conjunctiva sac of healthy subjects [34]. In this study, however, Actinobacteria were the third most abundant phylum in both groups, while firmicutes were the most abundant phylum. This relative abundance order was in line with the bacteria composition of the nasal mucosa [42], which receives drainage of tears from the conjunctival sac. While the relative abundance order was the same in both groups, there was statistically significant difference in the relative abundance of Proteobacteria, Bacteroidota and Cyanobacteria. However, whether this difference has clinical significance needs further investigation.

The Firmicutes/Bacteroidetes (F/B) ratio, which was a widely accepted indicator of dysbiosis in the gut microbiota, was also calculated in this study. Studies have shown that higher F/B ratio in the gut is associated with obesity, while lower F/B is usually observed in inflammatory status such as inflammatory bowel disease [43]. We are not sure if this could be applied to microbiome homeostasis on the conjunctival sac, but we calculated the F/B ratio in this study for both groups. The F/B ratio was lower in the AC group compared with that of the NC group, although the difference was not statistically significant. The altered F/B ratio on the conjunctival sac to some extent indicates the inflammatory status of the conjunctival sac in AC, but more potent data in further studies is needed to confirm this association.

In previous studies regarding the microbiome composition in healthy subjects, results were relatively consistent in the phylum level but showed variations at the genus level [44]. *Corynebacterium, Staphylococcus, Streptococcus* and *Propionibacterium* were detected consistently in all studies, with variances in the relative abundance. In this study, we also identified these genera in both the NC and AC groups. With higher *Acinetobacter* identified in the NC group but higher *Bacillus* detected in the AC group. At the species level, the difference of *Bacillus* and *Acinetobacter* was represented by the fact that there was a lot more abundant *Bacillus* in the AC group and more *Acinetobacter_guillouiae* in the NC group. Thus, we propose that a higher *B/A* ratio might be associated with allergic or inflammatory status of the conjunctival sac. The *B/A* ratio calculated at the genus level was significantly higher in AC than NC.

There have also been studies revealing that patients with vernal keratoconjunctivitis have more confluent colonies on culture, with *Staphylococcus* more commonly identified [45]. However, in this study, the relative abundance of *Staphylococcus* was similar in both groups. This might be explained by the more complex pathophysiology of vernal keratoconjunctivitis. While it also presents with inching and conjunctivital swelling, it is not simply the classic IgE-mediated hypersensitivity and Th2-mediated responses; other mechanisms including immunoglobulin G (IgG) mediated responses, basophil hypersensitivity and cellular delayed-typed hypersensitivity may be involved [46].

This study is a pilot study trying to discover if there is any dysbiosis in allergic conjunctivitis compared with normal controls. By analyzing the alpha diversity, beta diversity, microbiota composition and their relative abundance, we did figure out an alteration in the conjunctival sac microbiota. While we could not identify whether this alteration was a cause or a result, this paved a new way for us to better understand the pathophysiology of the disease and try to find more advanced and integrated treatment. 

This study has some limitations. Firstly, the clinical parameters only included sex and age, which did not show any differences in each subgroup. The subtype, severity and course of allergic conjunctivitis and its association with microbiome composition were not collected and further analyzed. Secondly, although the species accumulation curve demonstrated that sufficient samples were collected to represent the overall population. Further studies with larger sample size or in different geographical populations are still needed to confirm the consistency of these results.

## 5. Conclusions

The findings in this study demonstrated that the alpha diversity was not significantly changed in the AC group, except when evaluated with the Shannon index, which showed more diverse and richer microbiome composition in the AC group. The beta diversity was changed in the AC group and the microbiome relative abundance was different in AC group compared with that in the NC group. The dysbiosis might be correlated with inflammatory status of the conjunctival sac, and might provide further evidence to investigate the mechanism and treatment methods for allergic conjunctivitis.

## Figures and Tables

**Table 1:** Demographic characteristics of patients.

		AC	NC	*p-*Value
Sex	Male (*n* (%))	8 (28.57)	12 (30.77)	
	Female (*n* (%))	20 (71.43)	27 (69.23)	0.847
Age	≤18 (*n* (%))	2 (7.14)	2 (5.13)	
	19–35 (*n* (%))	10 (35.71)	26 (66.67)	
	36–60 (*n* (%))	16 (57.14)	9 (23.08)	
	>60 (*n* (%))	0 (0)	2 (5.13)	
	mean ± SD	31.69 ± 11.75	35.61 ± 11.03	0.847

AC: Allergic conjunctivitis; NC: Normal control; SD: Standard deviations.

**Table 2:** Relative abundance at the phylum level in AC and NC groups.

Phylum	Relative Abundance in NC Group (%)	Relative Abundance in AC Group (%)	*p-*Value
Firmicutes	45.52	41.72	0.333
Proteobacteria	29.23	39.15	0.018
Actinobacteriota	11.62	9.32	0.414
Bacteroidota	7.27	4.72	0.020
Cyanobacteria	3.13	1.90	0.038

NC: normal control, AC: allergic conjunctivitis.

## Data Availability

The data presented in this study are available on request from the corresponding author.

## Supplementary Materials

The following supporting information can be downloaded at: https://www.mdpi.com/article/10.3390/jcm11041130/s1, Figure S1: Rarefaction curves from 100 resamplings of each patient’s community at different sequencing depths; Figure S2: Species accumulation analysis showing the increase in OTUs detected with the addition of each sample.
