# Supplementary figures and images for: Characterization of Conjunctival Sac Microbiome from Patients with Allergic Conjunctivitis
Source: J Clin Med. 2022 Feb 21;11(4):1130. doi: 10.3390/jcm11041130 (PMC8875969; doi:10.3390/jcm11041130)

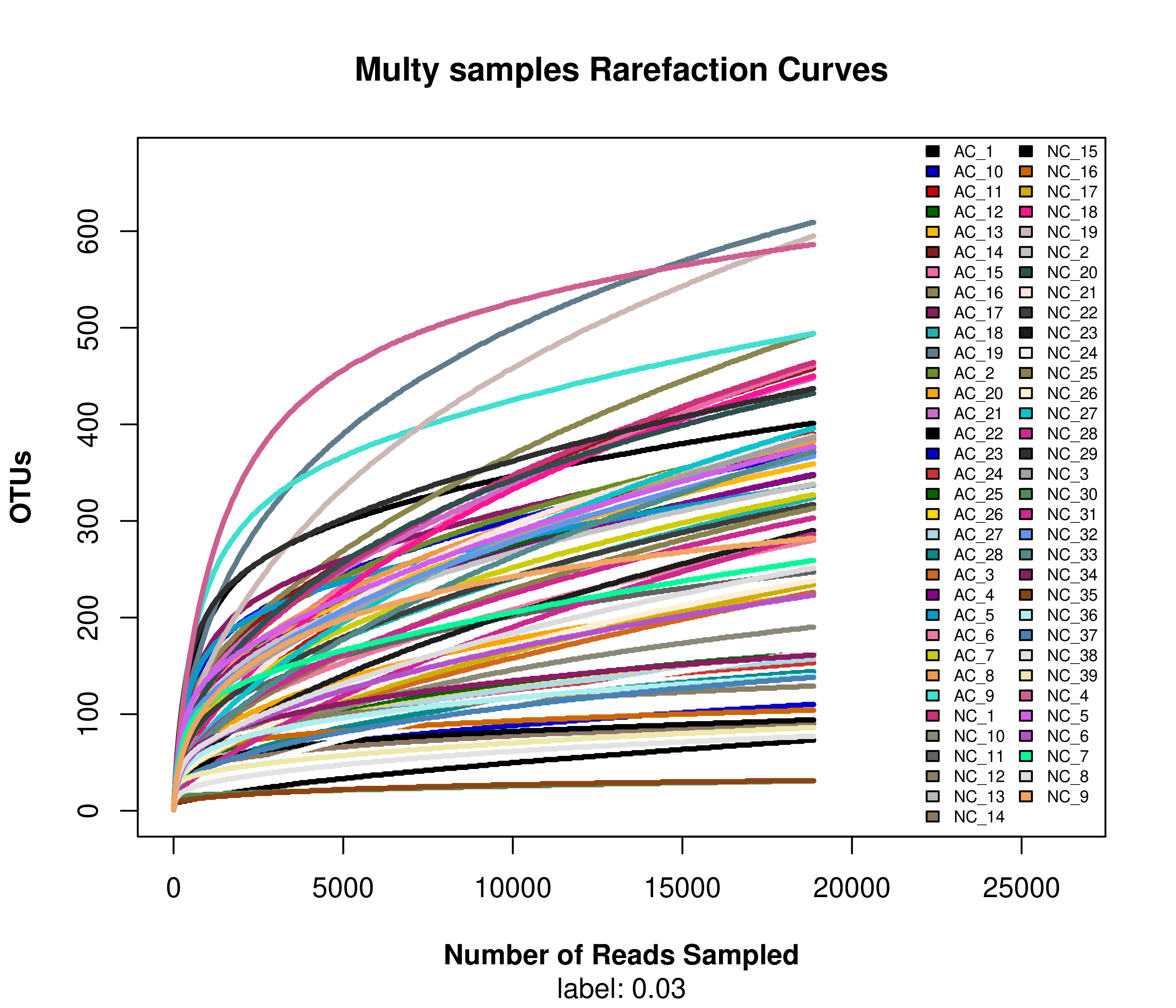

Supplement: Supplementary file 1 [file jcm-11-01130-s001.zip › Figure S1. Rarefaction curves from 100 resamplings of each patientí»s community at different sequencing depths.png]

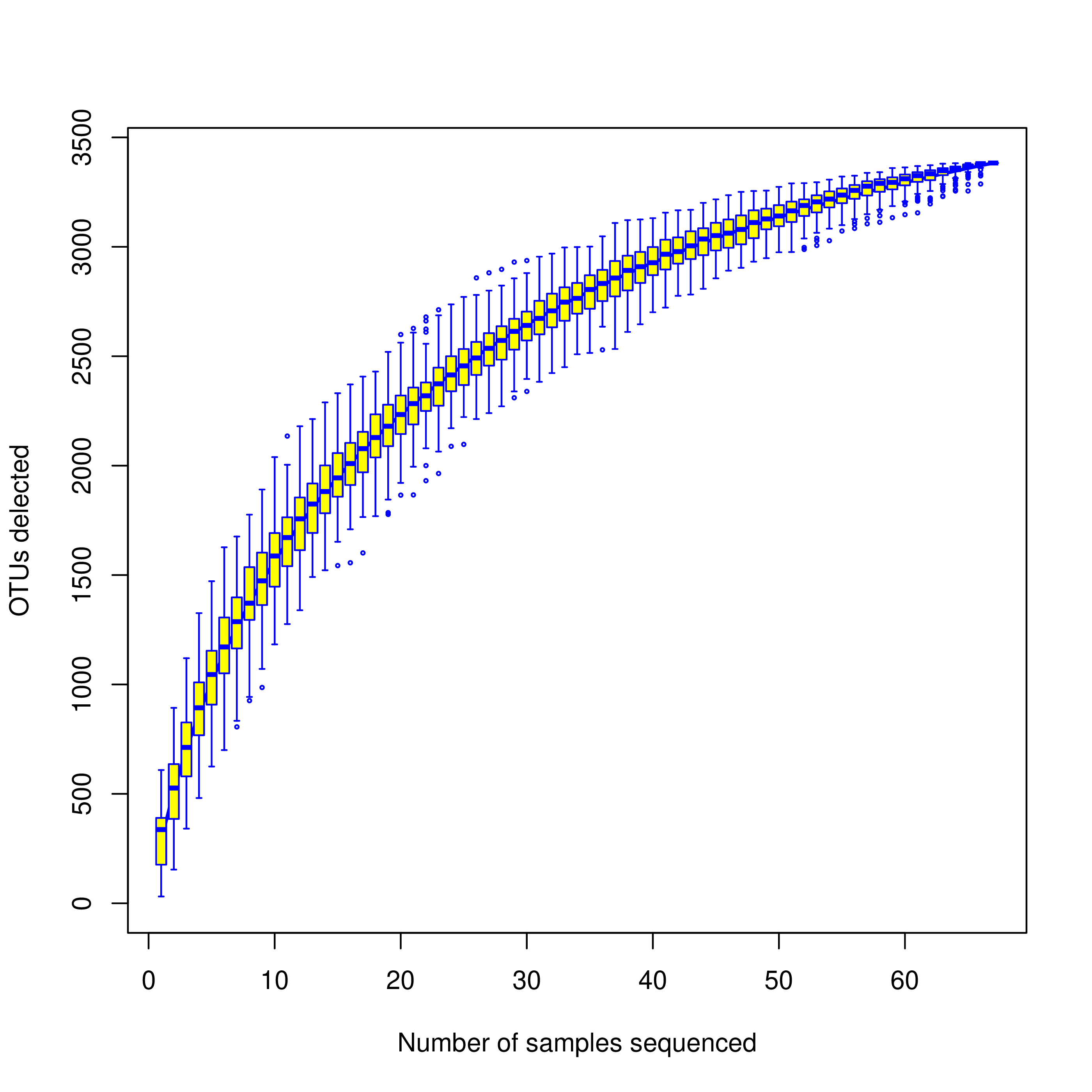

Supplement: Supplementary file 1 [file jcm-11-01130-s001.zip › Figure S2. Species accumulation analysis showing the increase in OTUs detected with the addition of each sample.png]
